# A computational study of base-catalyzed reactions of cyclic 1,2-diones: cyclobutane-1,2-dione

**DOI:** 10.3762/bjoc.9.64

**Published:** 2013-03-21

**Authors:** Nargis Sultana, Walter M F Fabian

**Affiliations:** 1 Department of Chemistry, University of Sargodha, Sargodha, Pakistan; 2 Institut für Chemie, Karl Franzens Universität Graz, Heinrichstr. 28, A-8010 Graz, Austria

**Keywords:** ab initio, density functional, reactive intermediate, rearrangement, ring opening

## Abstract

The reaction of cyclobutane-1,2-dione with hydroxide was studied by a variety of ab initio (MP2, SCS-MP2, CCSD(T), CEPA/1) and density functional (M06-2X) methods. Three possible reaction paths of the initially formed tetrahedral adduct leading to either 1-hydroxycyclopropane-1-carboxylate (benzilic acid type rearrangement, path A), α-oxobutanoate (path B) or γ-oxobutanoate (path C) were considered. Although the latter two products show similar or even more negative Gibbs free energies of reaction than calculated for the benzilic acid type rearrangement, the Gibbs free energies of activation are substantially higher. According to the calculations, the only feasible reaction appears to be the formation of 1-hydroxycyclopropane-1-carboxylate, which is corroborated by previous experimental observations.

## Introduction

**Scheme 1:**
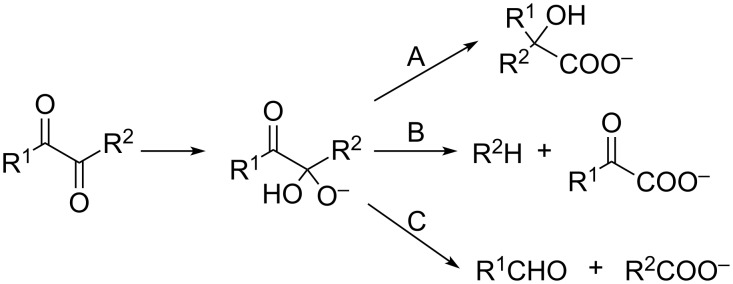
Reactions of 1,2-dicarbonyl compounds with base.

Addition of nucleophiles, e.g., OH^–^, to 1,2-dicarbonyl compounds leads to the formation of relatively stable tetrahedral adducts (Scheme 1) [1]. These adducts further react either by (i) fission of the R^2^–C bond and migration of R^2^ (benzil–benzilic acid rearrangement, path A); (ii) fission of the R^2^–C bond without migration of R^2^ resulting in α-oxocarboxylic acids (path B); or (iii) fission of the carbonyl-C–sp^3^-C with formation of an aldehyde and carboxylic acid (path C). In the case of benzils, depending on the substituents on the aryl rings, all three types of reactions have been observed [2].

**Scheme 2:**
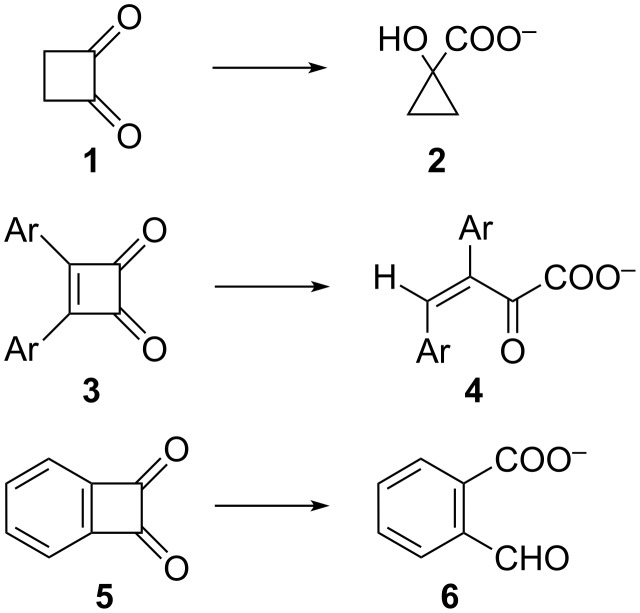
Reactions of cyclic 1,2-diones with base.

The benzilic acid rearrangement of cyclic 1,2-diones [3–4] leads to ring contraction, e.g., the rearrangement of cyclobutane-1,2-dione (**1**) to 1-hydroxycyclopropanecarboxylate (**2**) [5–7]. In contrast, cyclobut-3-ene-1,2-diones **3** react to 2-oxobut-3-enoates **4** (at least formally according to path B) [8], whereas benzocyclobutene-1,2-diones **5** lead to 2-formylbenzoates **6** (path C, Scheme 2) [9].

In view of the importance of the benzil–benzilic acid rearrangement in organic chemistry, several computational studies concerning this rearrangement [10–11] or related reactions (Favorskii rearrangement [12–14], halolactonisation [15]) have been published. To the best of our knowledge no attempt has been made so far to consider the additional pathways B and C in these reactions. Here we present a detailed computational study (DFT and ab initio) of the base-catalyzed reactions of cyclobutane-1,2-dione (**1**) taking into account all three possible pathways.

## Results and Discussion

**Scheme 3:**
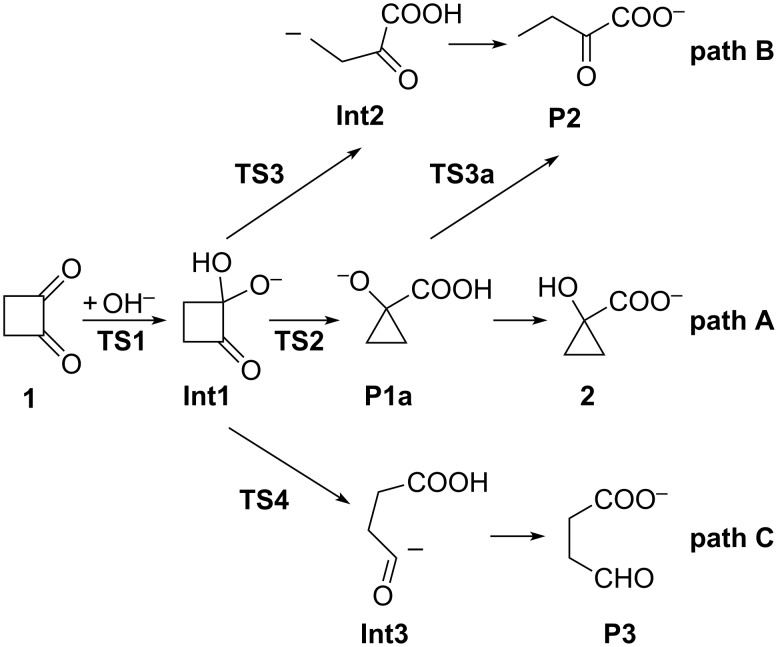
Possible intermediates, transition structures, and products considered for the reaction of cyclobutane-1,2-dione with hydroxide anion.

The various transition states, intermediates and products initially considered for the three reaction paths A, B, and C are depicted in Scheme 3. It turned out that not all of the structures shown in Scheme 3 could actually be located as stationary points on the potential-energy surface. On the other hand, some other stable as well as highly reactive intermediates and/or transition states were obtained (see below). Generally, in nucleophilic addition reactions to carbonyl compounds in aqueous solution, water not only acts as a solvent but frequently actively participates in the reaction, such as in water-assisted hydrolysis [16–20] or also in the benzil–benzilic acid rearrangement [11]. Therefore, cyclobutane-1,2-dione (**1**) hydrated by two water molecules, and hydroxide ion hydrated by four, i.e., **1**·(H_2_O)_2_ and [OH(H_2_O)_4_]^–^ , were used as reactants. Hence for all transition states, intermediates and products shown in Scheme 3, hydration by six water molecules is implied.

Relative Gibbs free energies with respect to the separated reactants **1**·(H_2_O)_2_ + [OH(H_2_O)_4_]^–^ including bulk aqueous solvation energies (SMD solvation model [21]) obtained by various computational procedures are collected in Table 1. More detailed results are provided in Supporting Information File 1. Before discussing the individual reaction paths in detail, a comparison of the computational procedures used (M06-2X [22], MP2 [23] and SCS-MP2 [24], the composite energy scheme *E*_C_ = *E*(MP2/6-311+G(2df,2p) + *E*{[CCSD(T) – MP2]/6-31+G(d)}; and LPNO-CEPA/1 [25–27]) is made.

### Comparison of computational procedures

Taking the CEPA/1 results as a reference, inspection of Table 1 reveals that all other computational procedures invariably lead to a greater stabilization of all stationary points considered with respect to the separated reactants. The largest deviation from the CEPA/1 energies is obtained with M06-2X, the smallest with SCS-MP2. The corresponding mean absolute deviations and RMS errors are 5.9, 6.3 (M06-2X); 3.6, 3.9 (MP2); 2.5, 2.9 (SCS-MP2); and 3.9, 4.0 (*E*_C_). However, the predicted trends agree very well with that obtained with CEPA/1 [Supporting Information File 1; the corresponding correlation coefficients *R*^2^ are 0.992 (M06-2X); 0.997 (MP2 and SCS-MP2); and 0.999 (*E*_C_)]. Inclusion of an empirical dispersion correction (DFTD3 [28]) in the M06-2X results further lowers the relative energies by ca. 0.8 kcal mol^–1^.

**Table 1:** Relative Gibbs free energies for all stationary points considered^a^.

	M06-2X	MP2	SCS-MP2	*E*_C_	CEPA/1

**IDC**	2.5	4.2	6.7	3.7	7.3
**TS1**	6.3	6.5	10.0	6.2	11.1
**Int1**	–6.1	–4.4	–4.1	–5.8	–2.2
**TS2**	7.7	6.9	9.2	9.4	14.3
**P1a**	–15.2	–10.3	–11.1	–9.0	–6.2
**2**	–32.6	–28.6	–29.9	–28.2	–24.4
**TS3a**	12.8	15.7	17.9	16.6	19.9
**P2**	–39.8	–37.5	–39.9	–39.4	–36.7
**TS4**	16.4	17.6	20.2	17.6	22.2
**Int4**	14.7	17.8	20.7	18.1	22.7
**TS5**	34.3	38.7	41.0	36.7	42.2
**P3a**	–29.3	–23.7	–23.1	–23.7	–21.2
**Int2a**	–25.5	–19.9	–20.9	–22.1	–18.4
**P2a**	–40.4	–37.3	–38.9	–38.6	–35.3

^a^Δ*G* (1 mol L^–1^ standard state, kcal mol^–1^) with respect to separated reactants **1**·(H_2_O)_2_ + [OH(H_2_O)_4_]^–^. All geometries optimized with M06-2X/6-31+G(d,p). For M06-2X, MP2, and SCS-MP2 single-point calculations the 6-311+G(2df,2p) basis set was used; LPNO-CEPA calculations were done with the def2-QZVPP basis set; *E*_C_ = *E*[MP2/6-311+G(2df,2p)] + *E*{[CCSD(T) – MP2]/6-31+G(d)}; Δ*G*_solv_ calculated by SMD M06-2X/6-31G(d).

**Figure 1:**
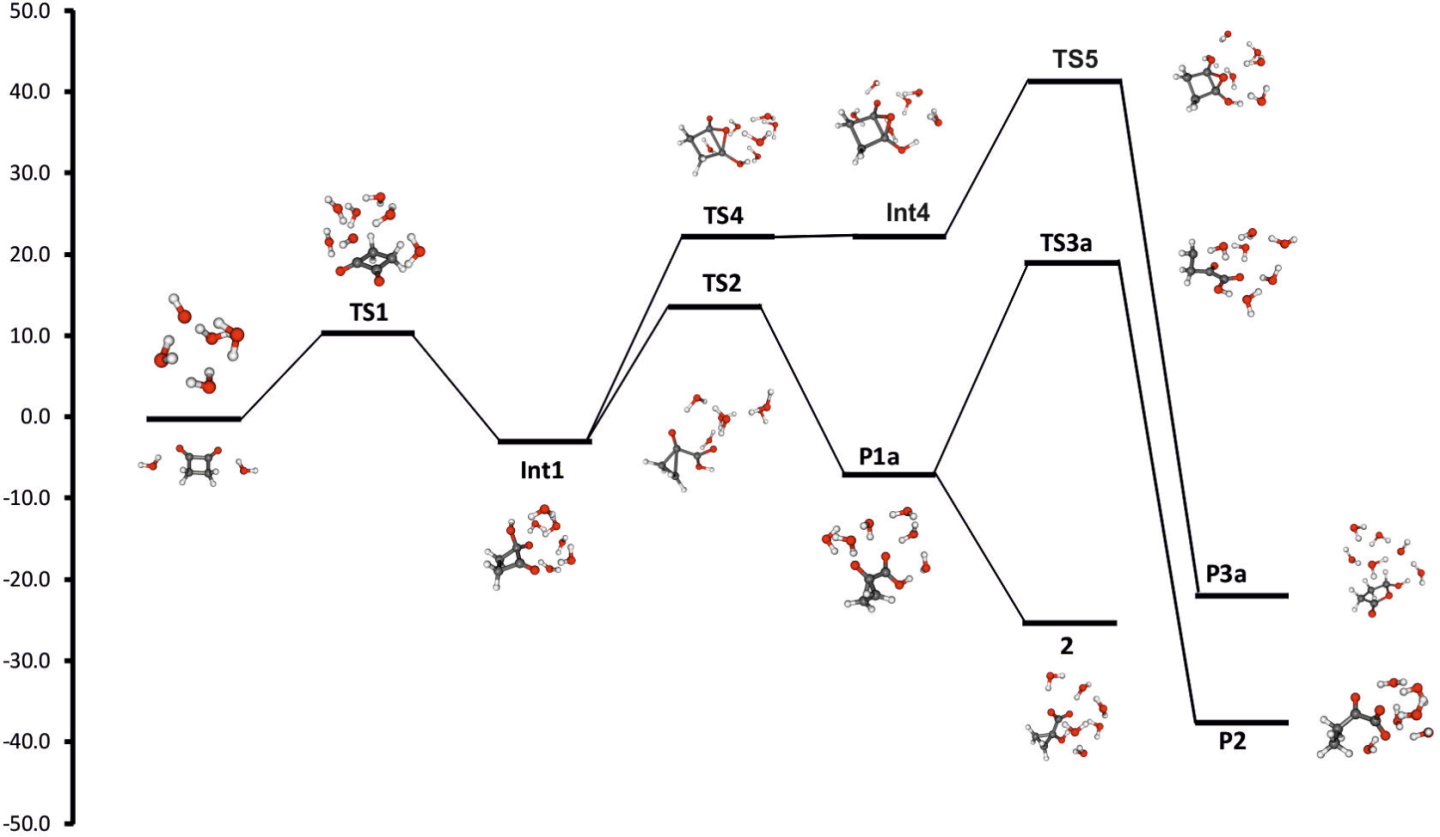
CEPA-1/def2-QZVPP calculated reaction paths for the reaction of **1**·(H_2_O)_2_ + [OH(H_2_O)_4_]^–^.

Figure 1 summarizes the energetic as well as structural aspects of all three possible reaction paths in the reaction of cyclobutane-1,2-dione with hydroxide anions.

### Individual reaction paths

**Figure 2:**
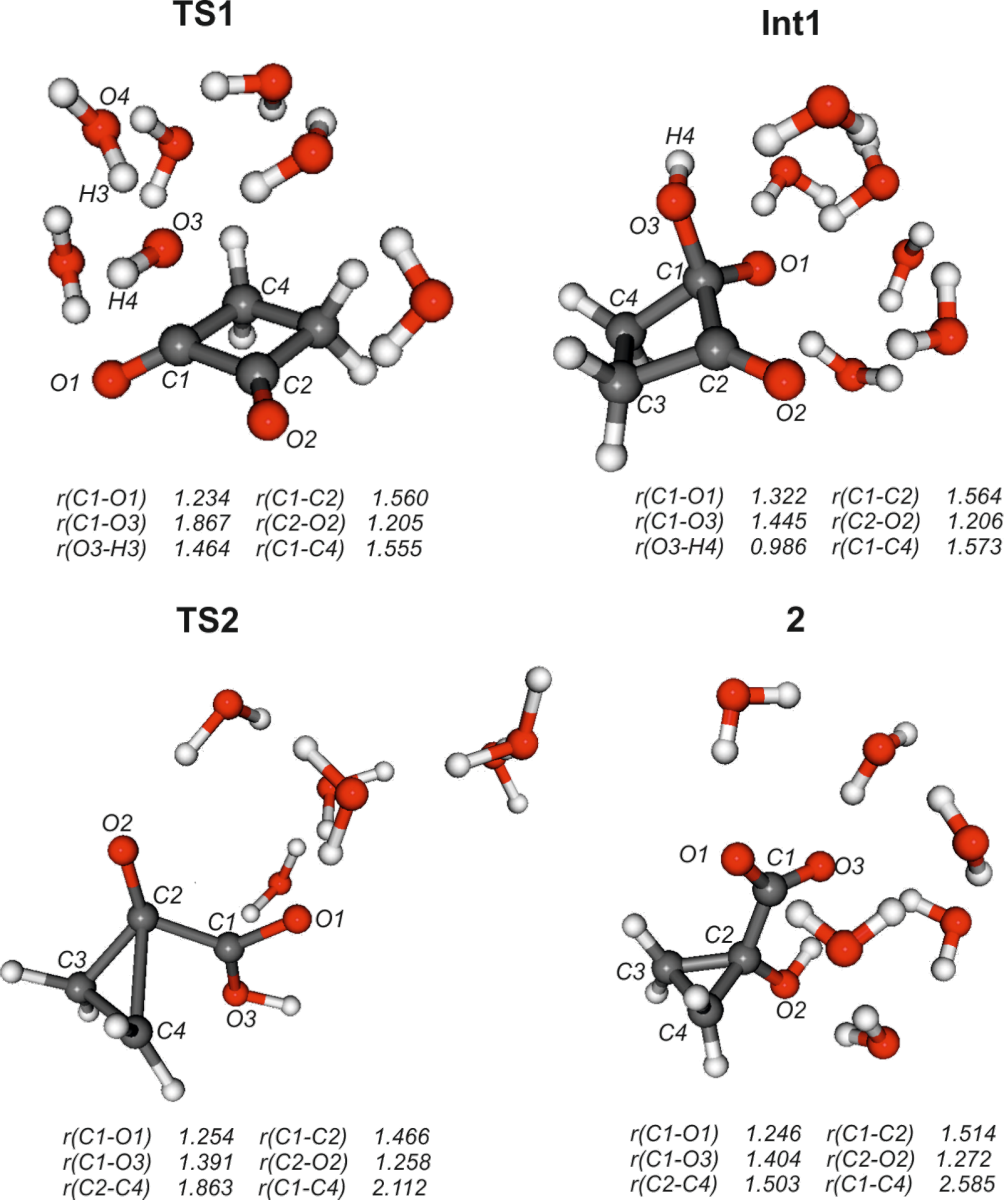
M06-2X/6-31+G(d,p) calculated structures of stationary points along the benzilic acid type rearrangement. Distances are given in angstroms (Å).

Addition of neutral nucleophiles to carbonyl compounds generally proceeds via formation of a prereaction complex; if Gibbs free energies instead of pure electronic energies or enthalpies are considered, such complexes usually become unstable [29]. In the present case without inclusion of bulk solvation effects (Supporting Information File 1) this prereaction complex (ion–dipole complex **IDC**) is quite stable with Δ*G* = –14 to –19 kcal mol^–1^ compared to the separated reactants. However, especially the large calculated [SMD-M06-2X/6-31G(d)] solvation energy of [OH(H_2_O)_4_]^–^ compared with that of the ion–dipole complex (Δ*G*_solv_ = –66 versus –54 kcal mol^–1^) results in an endergonic formation of this complex (Δ*G* = 2.5–7.3 kcal mol^–1^, Table 1). After passing transition state **TS1** (ν = 144 i cm^–1^), formation of the tetrahedral adduct by attack of [OH(H_2_O)_4_]^–^ at C1 leads to the first truly stable intermediate **Int1**. This intermediate is common to all further possible pathways. The nucleophile involved in the formation of **Int1** actually is a water molecule, since formation of the C1–O3 bond is accompanied by proton transfer H3 to O4 of the original hydroxide anion (Figure 2). An analogous concerted addition–proton transfer has also been calculated previously for the benzilic acid type rearrangements of biacetyl and benzil [11]. Car–Parrinello molecular dynamics simulations of the hydrolysis of formamide in basic solution indicated that the traditional view of attack by hydroxide anion rather than a first-solvation-shell water molecule is more likely; however, the more powerful electrophile methyl formate should react according to the general-base mechanism [30]. The second carbonyl group in 1,2-diones efficiently enhances the carbonyl reactivity to make attack by water the preferred mode of reaction.

#### Benzil–benzilic acid rearrangement (Path A)

With respect to intermediate **Int1**, the Gibbs free energy of activation of the benzilic acid rearrangement (fission of the C1–C4 and concerted formation of the C2–C4 bond) via **TS2** (ν = 246*i* cm^–1^) is ca. 15 kcal mol^–1^. The initial product **P1a** (1-carboxycyclopropanolate) is expected to easily convert to the final product 1-hydroxycyclopropanecarboxylate (**2**) by a simple acid–base equilibrium (protonation of the alcoholate, deprotonation of the carboxylic acid, Δ*pK_a_ ~* 10). Overall, path A is not only strongly exergonic [Δ*G*_react_ = –24.4 (CEPA/1) to –33 kcal mol^–1^(M06-2X)] but has also a quite low barrier [Δ*G*^≠^ = 6.9 (MP2) to 14.3 kcal mol^–1^(CEPA/1)], Table 1. Thus, this reaction should be quite feasible. Pertinent geometrical data are collected in Table S3 of Supporting Information File 1; M06-2X/6-31+G(d,p) optimized structures of relevant stationary points along path A are depicted in Figure 2.

The breaking C1–C4 bond is stretched from 1.573 Å in **Int1** to 2.112 Å in **TS2** (2.585 Å in **2**), while the newly formed bond C2–C4 is shortened from 2.125 Å in **Int1** to 1.863 Å in **TS2** (1.503 Å in **2**). The Wiberg bond indices resulting from an NBO analysis [31] for the C1–C4 bond are 0.92 (**Int1**) and 0.35 (**TS2**); those for the C2–C4 bond are 0.04 (**Int1**) and 0.40 (**TS2**), indicating nearly equal bond breaking and formation. The feasibility of the carbanion [1,2]-shift in the benzilic acid rearrangement has been attributed to the special shape [11] of the LUMO of 1,2-dicarbonyl compounds.

#### Path B

**Scheme 4:**
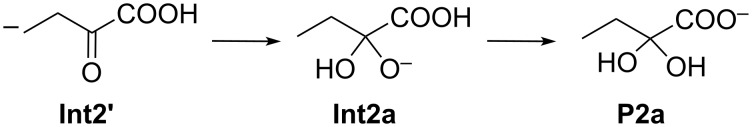
Reaction sequence calculated for an extended conformation of **Int2**.

Product **P2** should be even more stable than **2**. However, despite several attempts neither **TS3** nor **Int2** could be obtained. Instead, invariably **TS2** or **P1a** were obtained. It is tempting to assume that the relatively close contact between C2 and C4 preferentially results in C2–C4 bond formation rather than addition of a proton to **Int2**. To address this problem, optimization of an extended conformation **Int2’** was attempted. However, such a structure collapsed upon geometry optimization in a concerted proton transfer–nucleophilic addition reaction to intermediate **Int2a**. By a simple acid–base equilibrium (alcoholate–carboxylic acid → alcohol–carboxylate), this intermediate is expected to convert immediately to product **P2a**, i.e., the hydrate of product **P2** (Scheme 4).

**Figure 3:**
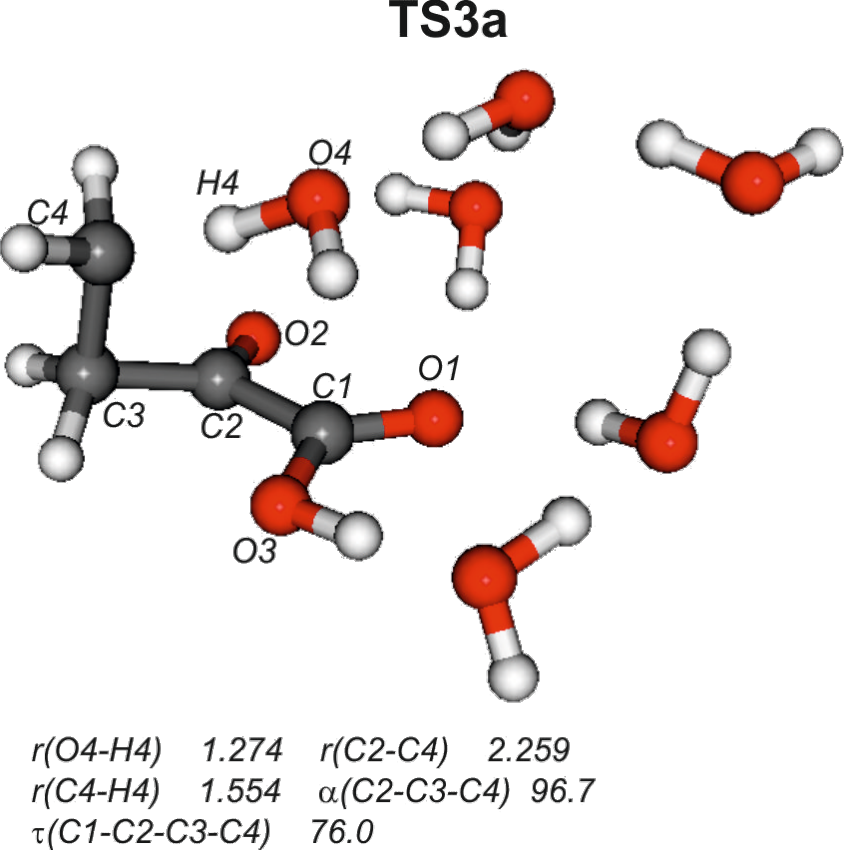
Calculated structure of transition state **TS3a**. Distances are given in angstrom (Å), angles in degrees.

While all attempts to locate transition state **TS3** as well as those ones leading to either **Int2’** or **Int2a** were unsuccessful, a path [**TS3a** (ν = 410*i* cm^–1^**,** Scheme 3 and Figure 3] directly connecting **P1a** instead of **Int1** with **P2**, could be obtained. Hence, path B actually does not start off from **Int1** but diverges at the initially formed product **P1a** of path A.

Compared with **TS2**, transition state **TS3a** (Figure 3) is characterized by significantly longer C1–C4 and C2–C4 bonds, 2.975 and 2.259 Å in **TS3a** versus 2.112 and 1.863 Å in **TS2**. Similarly, the C2–C3–C4 angle and the C1–C2–C3–C4 dihedral angle are much larger in **TS3a** (96.7° and 76.0°) than those found in **TS2** (76.6° and 54.8°), Table S3 in Supporting Information File 1. The main “movement” in **TS3a** is transfer of a proton from O4 to C4; consequently the imaginary frequency of **TS3a** is larger (ν = 410*i* cm^–1^) than that of **TS2** (ν = 246*i* cm^–1^) with C–C bond formation as the associated mode.

#### Path C

**Figure 4:**
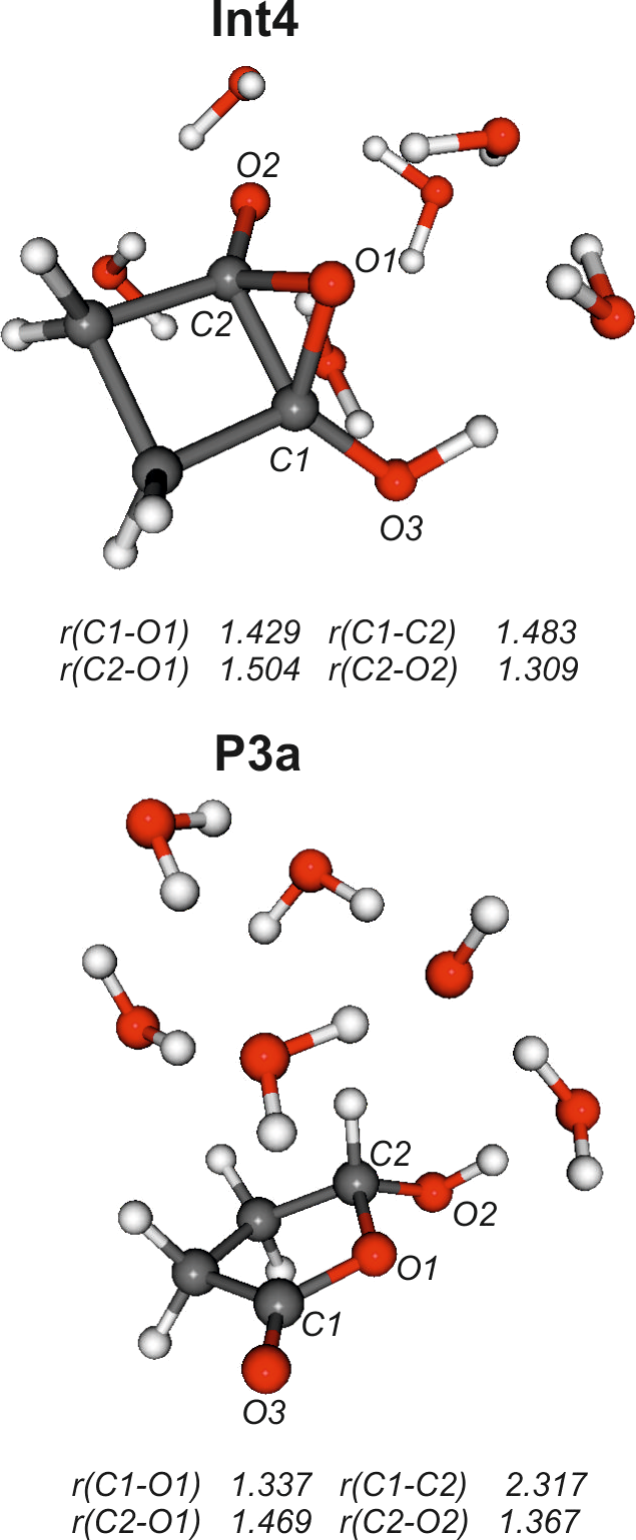
Calculated structures of pertinent stationary points along path C. Distances are given in angstroms (Å).

Similar to path B, all attempts to locate the initially proposed intermediate **Int3** were unsuccessful. Instead a more complicated pathway involving a high-energy bicyclic intermediate **Int4** was found (Figure 4).

**Scheme 5:**
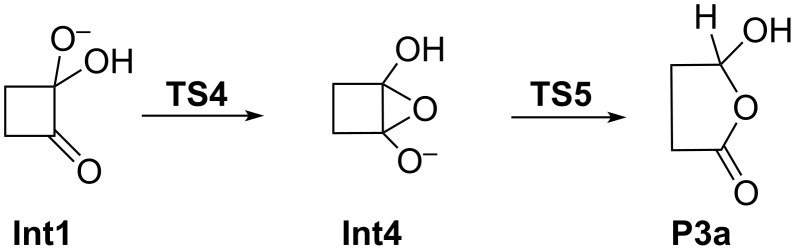
Actual path C obtained by the calculations (as in Scheme 3, **Int1**, **TS4**, **Int4**, and **TS5** are hydrated by six water molecules; **P3a** is the ion-dipole complex with [OH(H_2_O)_5_]^–^).

Furthermore, this intermediate did not react to product **P3** but instead through a concerted ring opening and proton transfer to **P3a**. The anticipated product **P3** of path C is a γ-oxocarboxylate. Such γ-oxocarboxylic acids or carboxylates are prone to ring–chain tautomerism [32–33]. Product **P3a** essentially is the ion-dipole complex between the ring tautomer of neutral γ-oxobutanoic acid with [OH(H_2_O)_5_]^–^ , and hence, its formation is completely reasonable. The actual pathway C obtained by the calculations is indicated in Scheme 5.

In fact, **Int4** is barely stable if Gibbs free energies and bulk aqueous solvation are taken into account (Table 1 and Supporting Information File 1) and should collapse more or less barrierless to the tetrahedral adduct **Int1**. In contrast, rearrangement to **P3a** involves a substantially higher barrier (40–45 kcal mol^–1^ with respect to **Int1**) than path A (ca. 15 kcal mol^–1^ with respect to **Int1**). **TS4**, **Int4** and **TS5** can be considered as bicyclic structures consisting of a 3-membered oxirane and a 4-membered cyclobutane ring. The two rings are inclined to each other as indicated by the angle α measured between the midpoints of the C3–C4 and C1–C2 bonds and the oxygen atom O1 [α = 103° (**TS4**), 106° (**Int4**), and 112° (**TS5**)]. The product **P3a** of path C has a largely planar five-membered ring structure (α = 165°). In both **TS4** and **Int4** the C1–C2 distance (1.48 Å) is in the range of C–C single bonds, while in **TS5** this bond is significantly stretched (*r =* 1.853 Å; in **P3a** this distance is *r* = 2.317 Å). In contrast the O1–C2 bond is shortened, i.e., 1.747 Å (**TS4**), 1.504 Å (**Int4**), and 1.385 Å (**TS5**), with a concomitant lengthening of the C1–O1 bond, i.e., 1.396 Å (**TS4**), 1.429 Å (**Int4**), and 1.531 Å (**TS5**). In the product **P3a** the proton of the O3–H group is transferred by involving the whole water chain to oxygen atom O2. This change in the position of the proton is accompanied by a shortening and lengthening, respectively, of the C1–O3 and C2–O2 distances: in **TS5 ***r*(C1–O3) = 1.383 Å and *r*(C2–O2) = 1.233 Å, while in **P3a ***r*(C1–O3) = 1.210 Å and *r*(C2–O2) = 1.367 Å. Finally, it should be noted that intermediates of the type **Int4** have been proposed [9] to be involved in the base-catalyzed reactions of benzocyclobutenediones (reaction **5** → **6** in Scheme 2).

## Conclusion

Ab initio (MP2, SCS-MP2, composite energy approach *E*_C_, LPNO-CEPA/1) and density functional methods (M06-2X) were applied to study the reaction of cyclobutane-1,2-dione in basic solution. The reaction system was modeled by using cyclobutane-1,2-dione hydrated with two water molecules and [OH(H_2_O)_4_]^–^ as the nucleophilic reagent. Three possible reaction pathways were considered, namely (i) a benzilic acid type rearrangement (path A); (b) ring-opening of the bond between an aliphatic carbon and that bearing the added OH^–^ group (path B); and (c) fission of the bond between the carbonyl carbon and that bearing the added OH^–^ group (path C). Attempts to locate path B starting directly from the tetrahedral intermediate **Int1** were unsuccessful. Instead, a reaction sequence diverging from the initially formed product **P1a** of path A was found. Path C involved transformations via high energy bicyclic transition states and/or intermediates. The final products of these latter two paths have comparable (path C) or even substantially more negative reaction energies (path B). However, the corresponding Gibbs free energies of activation are quite large. With respect to **Int1** these are at the CEPA/1 level 22.2 kcal mol^–1^ (path B; with respect to **P1a** Δ*G*^≠^ = 44.3 kcal mol^–1^) and 44.4 kcal mol^–1^ (path C). In contrast, path A is not only strongly exergonic but also has a significantly lower activation energy, Δ*G*_react_ = –22.2 kcal mol^–1^ and Δ*G*^≠^ = 16.5 kcal mol^–1^ with respect to **Int1**.

Hence, in agreement with experimental observations [5–7], cyclobutane-1,2-dione is calculated to react via benzilic acid rearrangement to the ring-contracted product, i.e., 1-hydroxycyclopropanecarboxylate (**2**).

## Computational details

Geometries were optimized by using the M06-2X density functional [22] and the 6-31+G(d,p) basis set [34–35] and characterized by frequency calculations as minima or transition states. For transition states, IRC calculations [36] were also done. These geometries were then used for M06-2X, MP2 [23] and SCS-MP2 [24] single-point calculations using the 6-311+G(2df,2p) basis set [37]. For coupled cluster CCSD(T) [38] and CEPA-1 [25–27] calculations the 6-31+G(d) and def2-QZVPP [39] basis sets, were used, respectively. Initial coordinates for [OH(H_2_O)_4_]^–^ were taken from the WATER27 subset of the GMTKN30 database [40–41] and reoptimized with M06-2X/6-31+G(d,p). [OH(H_2_O)_4_]^–^ was then placed about 6 Å above **1**·(H2O)_2_ and the combined system again optimized, resulting in the ion-dipole complex. An initial structure for **TS1** was obtained from a relaxed (i.e., optimization of all other coordinates) potential energy scan of the OH–carbonyl-carbon distance; this structure was then refined by transition-state optimization and further characterized by IRC calculations along both directions of the normal mode corresponding to the imaginary frequency. The final structures of both IRC calculations were then completely optimized. An analogous procedure, usually considering several possible reaction coordinates, was used for an initial guess of all other transition states. Bulk solvent effects (aqueous solution) were obtained by the SMD solvent model [21] at the M06-2X/6-31G(d) computational procedure. Frequencies obtained at the M06-2X/6-31+G(d,p) level are unscaled. Gibbs free energies are given relative to the separated reactants **1**·(H_2_O)_2_ and [OH(H_2_O)_4_]^–^ and contain a 1.9 kcal mol^–1^ correction for the standard state conversion 1 atm to 1 mol L^–1^ at *T* = 298.15 K. Dispersion corrections to the M06-2X results were added by Grimme’s DFTD3 procedure [28]. Programs used were ORCA [42], Gaussian 09 [43], GAMESS [44], and DFTD3 [45]; MOLDEN [46] and MOLEKEL [47] were used for structure building and visualization.

## Supporting Information

File 1 Detailed computational results and plot of MP2, SCS-MP2 and M06-2X vs. CEPA Δ*G*_rel_ values, pertinent structural data, and Cartesian coordinates of all stationary structures.
