# Supplementary material for: A computational study of base-catalyzed reactions of cyclic 1,2-diones: cyclobutane-1,2-dione
Source: Beilstein J Org Chem. 2013 Mar 21;9:594–601. doi: 10.3762/bjoc.9.64 (PMC3628910; doi:10.3762/bjoc.9.64)
Supplement: File 1 — Detailed computational results and plot of MP2, SCS-MP2 and M06-2X vs. CEPA ΔGrel values, pertinent structural data, and Cartesian coordinates of all stationary structures. [file Beilstein_J_Org_Chem-09-594-s001.pdf]

Supporting Information

for

A computational study of base-catalyzed reactions of cyclic 1,2-diones: cyclobutane-1,2-dione

Nargis Sultana[1,2] and Walter M. F. Fabian[2*]

Address: [1]Department of Chemistry, University of Sargodha, Sargodha, Pakistan and [2]Institut für Chemie, Karl Franzens Universität Graz, Heinrichstr. 28, A-8010 Graz, Austria

Email: Walter M. F. Fabian - walter.fabian@uni-graz.at

* Corresponding author

Detailed computational results and plot of MP2, SCS-MP2 and M06-2X vs. CEPA ΔG_{rel} values, pertinent structural data, and Cartesian coordinates of all stationary structures.

Table of Contents

Relative Gibbs free energies excluding bulk solvation S2

Relative electronic, ZPE and thermal, and solvation energies S3

Selected calculated structural parameters S4

Plot of M06-2x, MP2, SCS-MP2, E_C vs. LPNO-CEPA/1 relative Gibbs free energies S6

Cartesian coordinates of all stationary structures S7

Table S1: Relative Gibbs free energies excluding bulk solvation[a].

	M06-2X	MP2	SCS-MP2	E_C	CEPA/1
**IDC**	-19.2	-17.6	-15.1	-18.1	-14.4
**TS1**	-12.9	-12.7	-9.3	-13.0	-8.2
**Int1**	-25.3	-23.6	-23.4	-25.1	-21.4
**TS2**	-8.4	-9.3	-6.9	-6.8	-1.8
**P1a**	-33.9	-29.0	-29.7	-27.6	-24.9
**2**	-52.2	-48.2	-49.5	-47.8	-44.0
**TS3a**	-6.9	-4.0	-1.8	-3.0	0.2
**P2**	-63.4	-61.2	-63.6	-63.0	-60.3
**TS4**	-7.2	-6.0	-3.4	-6.0	-1.4
**Int4**	-9.0	-5.9	-3.0	-5.6	-1.0
**TS5**	10.6	15.0	17.4	13.0	18.5
**P3a**	-50.6	-45.0	-44.4	-45.0	-42.5
**Int2a**	-43.6	-38.0	-39.0	-40.2	-36.5
**P2a**	-59.6	-56.6	-58.2	-57.9	-54.6

[a] ΔG (kcal mol^{-1}) with respect to separated reactants **1**·$(H_2O)_2$ + $[OH(H_2O)_4]^-$. All geometries optimized with M06-2X/6-31+G(d,p). For M06-2X, MP2, and SCS-MP2 single point calculations the 6-311+G(2df,2p) basis set was used; LPNO-CEPA calculations were done with the def2-QZVPP basis set; $E_C = E$[MP2/6-311+G(2df,2p)] + E{[CCSD(T) – MP2]/6-31+G(d)}.

Table S2: Relative electronic, ZPE and thermal and solvation energies[a].

	M06-2X	MP2	SCS-MP2	E_C	CEPA	dG-corr	ΔG_{solv}
**IDC**	-35.2	-33.6	-31.1	-34.1	-30.4	17.9	21.7
**TS1**	-30.5	-30.3	-26.8	-30.6	-25.7	19.4	19.3
**Int1**	-44.4	-42.7	-42.5	-44.2	-40.5	21.0	19.2
**TS2**	-25.5	-26.3	-24.0	-23.9	-18.9	19.0	16.2
**P1a**	-50.7	-45.8	-46.6	-44.5	-41.7	18.8	18.6
**2**	-73.0	-69.1	-70.3	-68.6	-64.9	22.7	19.6
**TS3a**	-21.2	-18.2	-16.1	-17.3	-14.0	16.2	19.7
**P2**	-81.1	-78.9	-81.3	-80.7	-78.0	19.6	23.7
**TS4**	-28.6	-27.4	-24.8	-27.3	-22.7	23.3	23.6
**Int4**	-30.8	-27.7	-24.8	-27.4	-22.8	23.7	23.7
**TS5**	-9.7	-5.3	-2.9	-7.3	-1.8	22.2	23.7
**P3a**	-71.3	-65.7	-65.1	-65.7	-63.2	22.6	21.3
**Int2a**	-64.2	-58.6	-59.7	-60.9	-57.2	22.5	18.1
**P2a**	-80.7	-77.6	-79.3	-79.0	-75.7	23.0	19.3

[a]ΔG (kcal mol^{-1}) with respect to separated reactants **1**·$(H_2O)_2$ + $[OH(H_2O)_4]^-$. All geometries optimized with M06-2X/6-31+G(d,p). For M06-2X, MP2, and SCS-MP2 single point calculations the 6-311+G(2df,2p) basis set was used; LPNO-CEPA calculations were done with the def2-QZVPP basis set; E_C = E[MP2/6-311+G(2df,2p)] + E{[CCSD(T) – MP2]/6-31+G(d)}; dG-corr from unscaled M06-2X/6-31+G(d,p) frequencies and ZPE corrections; ΔG_{solv} calculated by SMD M06-2X/6-31G(d).

Table S3: Selected calculated structural parameters[a].

	1·$(H_2O)_2$	TS1	Int1	TS2	2
C1-O1	1.198	1.234	1.322	1.254	1.246
C1-C2	1.572	1.560	1.564	1.466	1.514
C2-O2	1.198	1.205	1.206	1.258	1.272
C1-O3	-	1.867	1.445	1.391	1.404
C2-C3	1.533	1.537	1.528	1.505	1.496
C1-C4	1.533	1.555	1.573	2.112	2.585
C2-C4	2.188	2.179	2.125	1.863	1.503
C2-C3-C4	90.4	89.8	87.2	76.6	59.6
C1-C2-C3-C4	0.0	-2.6	16.7	54.8	-109.8
	**TS4**	**Int4**	**TS5**	**P3a**	**TS3a**
C1-O1	1.396	1.429	1.531	1.337	1.224
C1-C2	1.484	1.483	1.853	2.317	1.545
C2-O2	1.261	1.309	1.233	1.367	1.222
C1-O3	1.383	1.369	1.383	1.210	1.314
C2-C3	1.535	1.541	1.531	1.527	1.486

C1-C4	1.536	1.522	1.520	1.518	2.975
C2-C4	2.162	2.177	2.306	2.399	2.259
C2-O1	1.747	1.504	1.385	1.469	-
C2-C3-C4	88.5	88.9	97.2	103.6	96.7
C1-C2-C3-C4	-8.3	-2.7	9.5	14.0	76.0

[a]M06-2X/6-31+G(d,p) results; distances in Å, angles in degrees.

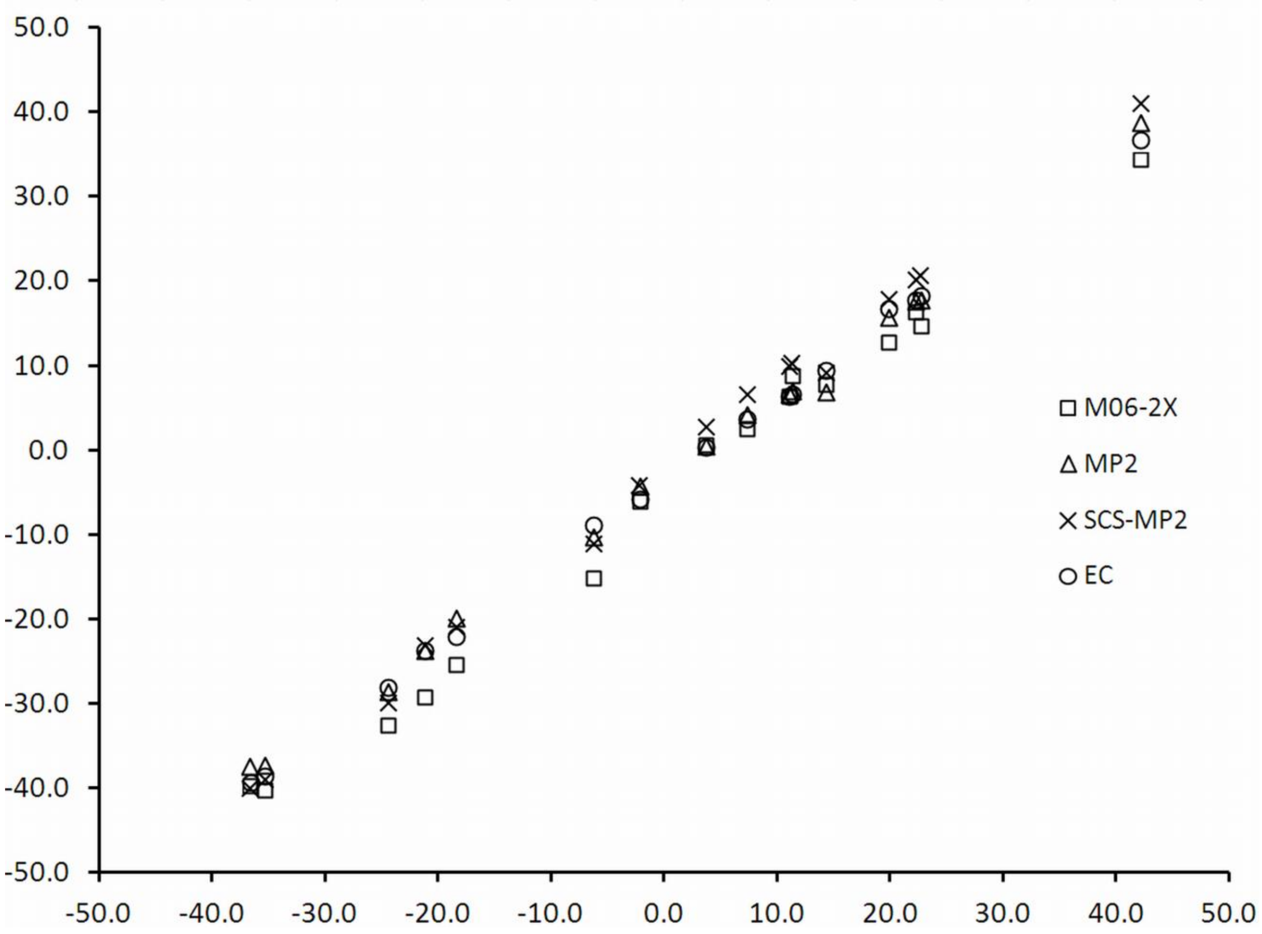

Figure S1: Plot of M06-2x, MP2, SCS-MP2, and E_C vs. LPNO-CEPA/1 relative Gibbs free energies.

cyclobutanedione + $2H_2O$: E(RM062X/6-31+G(d,p)) = -457.928387622

C	-0.77498	-1.01790	0.00042
C	0.77498	-1.01790	-0.00039
C	0.78605	0.51497	-0.00029
O	1.62517	1.37058	-0.00049
C	-0.78605	0.51497	0.00030
O	-1.62517	1.37058	0.00049
H	-1.26439	-1.43703	-0.88185
H	-1.26323	-1.43660	0.88356
H	1.26322	-1.43661	-0.88353
H	1.26439	-1.43702	0.88188
O	-3.67859	-0.60460	-0.00040
H	-4.63743	-0.53620	0.00001
H	-3.33272	0.29957	0.00010
H	3.33272	0.29957	-0.00008
O	3.67859	-0.60460	0.00035
H	4.63743	-0.53619	0.00003

$[OH(H_2O)4]^-$:   E(RM062X/6-31+G(d,p)) = -381.497273531

O	-0.06850	-0.69264	2.04151
O	-1.50909	-0.34609	0.00000
H	1.35811	-0.57488	0.77137
H	-0.06284	0.20017	2.40277
O	1.88015	-0.28094	0.00000
H	1.35811	-0.57488	-0.77137
O	-0.06850	-0.69264	-2.04151
H	-0.06284	0.20017	-2.40277

H	-0.73170	-0.63228	-1.25021
O	-0.06850	1.99353	0.00000
H	0.74908	1.46976	0.00000
H	-0.76781	1.30269	0.00000
H	-0.73170	-0.63228	1.25021
H	-2.43295	-0.60830	0.00000

IDCa: E(RM062X/6-31+G(d,p)) = -839.479243669

H	3.43390	0.01694	0.59990
O	3.36539	0.69255	1.29692
H	2.76683	1.34320	0.89476
O	1.59417	2.13122	-0.47223
H	2.24169	2.35414	-1.14876
O	0.38802	0.07173	-1.12609
H	0.19178	-0.06305	-2.06149
H	-1.10211	1.14844	-0.92430
O	-1.86653	1.75043	-0.79974
H	-1.48959	2.59314	-0.49571
H	0.49451	3.33384	-0.01401
O	-0.26931	3.90418	0.25102
H	-0.29537	3.87439	1.21171
H	-1.07977	0.33017	1.11501
H	-1.70008	-1.01393	2.10311
C	-1.01090	-0.74217	1.29876
C	0.42455	-1.31897	1.40747
H	0.60467	-1.95399	2.28037
H	1.24063	-0.59867	1.31140

C	0.18911	-2.16003	0.14375
O	0.80427	-3.01623	-0.43302
C	-1.25761	-1.58108	0.03408
O	-2.20878	-1.82224	-0.65876
H	1.07520	1.26706	-0.81477
O	2.88209	-0.98331	-0.96511
H	2.73390	-1.92716	-0.83151
H	1.96634	-0.61279	-1.06765
O	-3.95413	0.30979	0.46087
H	-3.70638	-0.52640	0.04460
H	-3.32855	0.94324	0.06219

TS1: E(RM062X/6-31+G(d,p)) = -839.473374959

H	3.100064	0.719273	1.252814
O	2.926497	1.675043	1.300951
H	2.840287	1.900668	0.362548
O	1.820094	1.464806	-1.371798
H	1.876373	1.824700	-2.262100
O	-0.106392	-0.132378	-1.238091
H	-0.263459	-0.637566	-2.046976
H	-1.393226	1.089227	-0.925841
O	-2.021298	1.769106	-0.601626
H	-1.482264	2.305004	0.001800
H	0.788720	2.581957	-0.162992
O	0.336360	2.899857	0.637671

H	0.918950	2.593425	1.347946
H	-1.728899	0.216558	1.307053
H	-1.566399	-1.237514	2.323335
C	-1.209548	-0.737528	1.418399
C	0.326988	-0.697494	1.227719
H	0.890358	-1.241849	1.990982
H	0.760135	0.291967	1.085315
C	0.202382	-1.551699	-0.065792
O	0.996167	-2.356171	-0.561458
C	-1.335415	-1.638802	0.180180
O	-2.262876	-2.188283	-0.358315
H	1.032245	0.779047	-1.364991
O	3.266166	-0.772521	-0.144923
H	2.613192	-1.495038	-0.244641
H	2.982148	-0.117836	-0.801145
O	-4.252908	0.098772	0.141848
H	-3.826459	-0.749465	-0.040853
H	-3.593162	0.769907	-0.107977

IDC: E(RM062X/6-31+G(d,p)) = -839.483915125

H	-3.46174	0.31211	-1.31075
O	-3.21337	1.24597	-1.25999
H	-2.93364	1.32037	-0.32308
O	-2.00989	1.00450	1.22257
H	-2.38838	1.43497	1.99568
O	0.27695	0.09147	1.55402
H	0.33692	-0.46473	2.33720

H	1.45953	1.48467	1.22338
O	1.93021	2.20738	0.76425
H	1.23050	2.59604	0.21199
H	-1.10818	2.10411	0.24253
O	-0.60276	2.58150	-0.46287
H	-1.12722	2.42703	-1.25891
H	1.25359	0.67753	-0.97634
H	1.60316	-0.52624	-2.24609
C	1.08664	-0.35162	-1.29822
C	-0.38783	-0.81712	-1.22149
H	-0.79938	-1.27609	-2.12529
H	-1.08833	-0.06397	-0.85543
C	-0.01359	-1.86292	-0.16486
O	-0.59662	-2.74730	0.40304
C	1.47863	-1.38451	-0.23523
O	2.50740	-1.74621	0.26753
H	-0.70802	0.46480	1.49570
O	-3.24186	-1.25246	0.28481
H	-2.60028	-1.97230	0.34403
H	-2.83095	-0.49652	0.76843
O	4.06923	0.76671	-0.48990
H	3.80203	-0.11113	-0.18734
H	3.42299	1.37391	-0.08444

TS1a: E(RM062X/6-31+G(d,p)) = -839.473184876

C	2.39124	-1.91915	0.03815
C	2.17884	-0.50976	-0.54820

O	2.48857	0.02200	-1.58940
C	1.83786	-1.42887	1.40262
C	1.60530	-0.02402	0.80129
O	1.39920	1.06079	1.30189
O	0.10758	3.47735	0.49336
O	0.20045	1.83895	-1.79453
O	-0.03004	-0.79210	-0.30526
O	-1.69179	-2.65326	-0.97310
O	-2.15685	1.71991	-0.01142
O	-3.77518	-0.84310	-0.30023
O	-1.73180	-0.45556	1.74473
H	3.44837	-2.20120	0.04573
H	1.79781	-2.67964	-0.46919
H	0.92690	-1.90994	1.75765
H	2.58278	-1.39288	2.20298
H	-2.55348	-0.81168	1.37033
H	-1.04361	-0.63600	1.05320
H	-1.49355	1.55224	-0.70062
H	-1.94275	1.10990	0.72492
H	-0.96689	-1.97698	-0.77027
H	-1.71009	-3.24206	-0.21221
H	-3.46930	0.05752	-0.48676
H	-3.13971	-1.44843	-0.72849
H	-0.19280	-0.05684	-0.91478
H	-0.82021	3.19809	0.55950
H	0.60642	2.76853	0.93458
H	0.35061	2.56226	-1.15635

H	1.05890	1.39809	-1.90167

Int1: E(RM062X/6-31+G(d,p)) = -839.500175556

C	-2.19657	-1.04298	-1.51195
C	-1.22543	-0.07515	-0.74019
O	0.08136	-0.26771	-0.79607
C	-3.18639	-1.03482	-0.31650
C	-2.04360	-0.47798	0.53060
O	-1.77721	-0.56345	1.70324
O	0.98344	-0.41934	2.60461
O	1.38794	-2.31773	0.47650
O	-1.60761	1.29164	-1.00954
O	-0.56328	3.15610	1.03309
O	3.62818	-0.77638	-0.20227
O	2.27378	0.58396	-2.36779
O	1.64220	1.35550	0.44542
H	-1.71833	-2.01566	-1.64813
H	-2.57316	-0.65649	-2.46039
H	-3.97341	-0.28429	-0.43685
H	-3.61375	-1.97997	0.02394
H	2.08703	1.75900	-0.31379
H	0.98166	0.71816	0.00412
H	3.01392	-1.52046	-0.04103
H	3.22687	-0.06137	0.31512
H	-1.13974	2.61295	0.47626
H	0.27890	2.67310	1.00953
H	2.92756	0.05892	-1.87470

H	1.42395	0.20575	-2.07760
H	-0.97969	1.58970	-1.68342
H	1.40727	0.31327	2.13215
H	0.03606	-0.31928	2.41377
H	1.30228	-1.92760	1.36413
H	0.83412	-1.72086	-0.07238

TS2: E(RM062X/6-31+G(d,p)) = -839.468667489

C	-3.08007	-1.02966	-1.15601
C	-1.44060	0.19019	-0.62137
O	-0.24497	-0.01946	-0.93551
C	-3.67837	-0.44841	0.09249
C	-2.20836	-0.51649	0.40829
O	-1.70806	-1.38866	1.16402
O	0.84654	-1.35056	2.10263
O	1.56013	-2.06902	-0.42001
O	-1.99534	1.39064	-1.05231
O	-0.51465	3.41380	0.64836
O	3.93614	-1.22810	-1.01952
O	3.93858	1.35737	-0.26174
O	1.37068	1.25771	0.93377
H	-2.92049	-2.10187	-1.18204
H	-3.20614	-0.51371	-2.10161
H	-4.11294	0.54053	-0.03056
H	-4.25717	-1.10572	0.73606
H	2.29485	1.37312	0.64414
H	0.92303	0.78423	0.20293

H	3.00280	-1.55855	-0.82069
H	4.53070	-1.83648	-0.57213
H	-1.24098	2.85368	0.34663
H	0.20070	2.78882	0.86398
H	4.00396	0.40987	-0.53260
H	3.83984	1.85633	-1.07778
H	-1.37313	1.71265	-1.72040
H	1.09841	-0.41625	2.01824
H	-0.10397	-1.37593	1.83712
H	1.41249	-1.97962	0.55122
H	0.83999	-1.51483	-0.78034

P1a: E(RM062X/6-31+G(d,p)) = -839.509427431

C	1.15567	2.37550	1.24643
C	-0.49995	1.10389	-0.21594
O	-0.83275	0.03496	-0.71594
C	1.27961	2.91997	-0.14711
C	0.92995	1.44730	0.05601
O	1.82054	0.48994	-0.23135
O	3.12732	-1.27698	-1.83320
O	2.83835	-1.18316	1.34160
O	-1.39915	2.03027	0.12518
O	-3.91174	1.02184	-0.15473
O	0.74650	-2.26848	-0.11198
O	-1.51106	-3.01200	1.14701
O	-3.13415	-1.48777	-0.52690
H	2.06939	2.15339	1.78713

H	0.30478	2.69326	1.83943
H	0.51255	3.60591	-0.49191
H	2.27683	3.06190	-0.54893
H	-2.80603	-2.12737	0.13577
H	-2.30907	-1.06146	-0.82488
H	1.48666	-2.09137	0.50967
H	0.52312	-1.37208	-0.43562
H	-4.30257	1.23492	-1.00752
H	-3.76958	0.03944	-0.16400
H	-0.66471	-2.81461	0.66449
H	-1.37895	-2.64187	2.02503
H	-2.31501	1.66268	0.02315
H	2.45903	-1.94406	-1.62918
H	2.72346	-0.46089	-1.46853
H	3.61731	-1.40987	0.81951
H	2.43015	-0.39683	0.81256

P1b = 2: E(RM062X/6-31+G(d,p)) = -839.54451899

C	2.76019	-1.80292	-0.04742
C	2.63687	-0.65933	0.92652
H	3.46711	0.03708	0.98753
H	2.06986	-0.81577	1.83731
H	1.54747	0.36007	-1.93722
C	1.86874	-0.63234	-0.35638
O	2.29784	0.29120	-1.32242
H	3.66940	-1.86228	-0.63601
H	2.27388	-2.73867	0.20536

C	0.37205	-0.85604	-0.33163
O	-0.26460	-0.30631	-1.28617
O	-0.10426	-1.54488	0.59125
H	-1.68407	-2.69072	0.53140
O	-2.57294	-3.04115	0.35464
H	-3.00433	-2.28652	-0.07075
H	-3.29110	-0.20581	-1.74777
O	-2.90055	-0.43597	-0.89953
H	-1.91621	-0.46685	-1.05353
H	-2.89520	1.00922	0.40287
O	-2.58179	1.82417	0.83201
H	-2.07825	2.27117	0.12446
O	-0.74876	2.52631	-1.19987
H	0.02171	2.76659	-0.64955
H	-0.61396	1.57451	-1.35271
H	1.98310	1.85103	-0.05757
O	1.48880	2.46599	0.50556
H	1.00026	1.90708	1.14692
O	-0.23996	0.93495	2.10643
H	-0.26040	-0.00358	1.85800
H	-1.10645	1.28280	1.80930

P2: E(RM062X/6-31+G(d,p)) = -839.555289901

C	1.03668	-3.25686	-0.60922
C	2.06469	-2.14316	-0.40310
C	1.51109	-0.75873	-0.64358
O	2.20148	0.15290	-1.06201

C	0.02441	-0.51880	-0.32021
O	-0.29512	-0.55836	0.89440
O	-0.71385	-0.30653	-1.30956
H	2.41710	-2.13624	0.63756
H	2.93828	-2.25721	-1.05178
H	1.48921	-4.23272	-0.41471
H	0.19182	-3.13502	0.07489
H	0.64581	-3.25644	-1.63107
O	-1.43750	2.40927	-0.94380
H	-1.13851	1.55277	-1.29591
H	-1.86650	2.15858	-0.10201
O	1.32941	2.80577	-0.29227
H	1.65192	2.01080	-0.74787
H	0.38661	2.88080	-0.52682
O	-3.49182	0.09652	-1.51796
H	-2.58178	-0.25878	-1.48567
H	-3.36617	1.01107	-1.80135
O	1.91136	-1.10833	2.69596
H	1.05387	-1.15533	2.24306
H	2.07106	-0.15044	2.71449
O	1.19542	1.64239	2.27716
H	0.42100	1.12614	2.01033
H	1.38408	2.18285	1.48677
O	-2.68806	0.92318	1.13077
H	-1.92981	0.30922	1.18564
H	-3.26334	0.52999	0.45515

Int4: E(RM062X/6-31+G(d,p)) = -839.468068741

C	-2.830678	-1.321073	0.321440
C	-1.924857	-0.098541	0.333066
O	-1.894578	0.409656	-1.001658
C	-0.964913	-0.708837	-0.618031
C	-1.866099	-1.953199	-0.741087
O	-1.828613	0.791445	1.369273
O	0.323633	-0.602772	-0.819501
H	-2.291633	-2.111883	-1.735716
H	-1.370873	-2.861250	-0.385546
H	-2.823342	-1.859952	1.272684
H	-3.858444	-1.131261	-0.004720
H	-1.612636	1.681922	0.989699
O	1.590635	-2.683830	0.594394
O	1.058610	2.121007	-1.005232
O	0.986332	-0.086449	1.726119
O	3.137186	-0.914591	-1.085695
O	3.217278	1.330096	0.667360
O	-1.308594	3.030756	-0.063008
H	1.863370	2.055272	-0.457350
H	0.747406	1.196781	-1.100702
H	2.580092	0.954690	1.299768
H	3.426897	0.585526	0.073336
H	2.240674	-0.704957	-1.398007
H	2.981088	-1.673036	-0.498815
H	0.158833	0.277325	2.074484
H	0.794531	-0.192583	0.755616

H	1.027832	-2.185174	-0.031183
H	1.529611	-2.139395	1.393850
H	-0.363878	2.888835	-0.308844
H	-1.765371	2.546484	-0.767312

TS5: E(RM062X/6-31+G(d,p)) = -839.443477678

C	-2.64240200	0.63808500	0.69785300
C	-1.15405000	0.33027300	0.70984200
O	-0.79826500	0.72882800	-0.72517500
C	-1.45125000	-0.47483200	-0.93282800
C	-2.91241300	-0.24867100	-0.53576700
O	-0.31705000	1.00233500	1.58223900
O	-0.97921600	-1.40401900	-1.59186300
H	-3.44816500	0.25340500	-1.35045700
H	-3.40064900	-1.19811000	-0.30372600
H	-3.14340900	0.29367700	1.60616800
H	-2.88398200	1.70227000	0.52663600
H	-0.26470100	1.94404000	1.30751400
O	-0.81362400	-2.86535500	0.88315700
O	1.79800100	1.74136100	-1.19974000
O	2.01920400	-0.49636400	1.80078500
O	1.53871000	-2.10516600	-0.58957100
O	3.74404400	-0.15618200	-0.35768200
O	-0.08250500	3.36616900	0.04439500
H	2.58940400	1.21819500	-0.97580900
H	1.04865600	1.12579200	-1.12395000
H	3.41122500	-0.08594700	0.55713000

H	3.28245000	-0.94557900	-0.68139100
H	0.82827600	-1.70707800	-1.13186700
H	1.03921100	-2.72528300	-0.03221000
H	1.27840300	0.13457200	1.86778800
H	1.71772600	-1.11579900	1.11641300
H	-1.18149600	-2.92110800	-0.01029900
H	-0.86969100	-1.89813600	1.05437600
H	0.78292800	3.05199500	-0.28614400
H	-0.69988900	2.91111100	-0.54712300

P3a: E(RM062X/6-31+G(d,p)) = -839.543187140

C	-1.518598	-0.924722	1.583360
C	-1.227184	0.372347	0.832915
O	-1.784152	0.154442	-0.508987
C	-1.922217	-1.149882	-0.769419
C	-1.618475	-1.967358	0.472613
O	-1.834497	1.450690	1.412734
O	-2.231262	-1.567110	-1.862433
H	-2.397178	-2.720147	0.610958
H	-0.667219	-2.485481	0.316443
H	-0.728979	-1.144727	2.302813
H	-2.471969	-0.793067	2.104336
H	-1.508817	2.241071	0.908781
O	1.496742	-1.761385	1.046617
O	1.297301	1.809640	-0.835776

O	2.781482	0.910417	1.214943
O	1.066668	-0.590482	-1.392778
O	3.823366	-1.028454	-0.604852
O	-0.839058	3.097626	-0.418481
H	1.898376	2.267953	-1.432224
H	1.128759	0.458491	-1.228890
H	3.711081	-0.265145	-0.006575
H	3.044588	-0.975351	-1.182625
H	0.359567	-0.754434	-2.026339
H	1.227453	-1.399149	0.172362
H	2.227827	1.360467	0.523426
H	2.224995	0.190791	1.547326
H	2.424493	-1.987584	0.885656
H	-0.156978	0.542192	0.662682
H	0.064565	2.627537	-0.593044
H	-1.411051	2.773687	-1.123725

TS4: E(RM062X/6-31+G(d,p)) = -839.474534165

C	2.456452	-1.424898	-0.864228
C	1.921303	-2.482084	0.155263
C	0.676624	-1.600303	0.331892
O	-0.015715	-1.596053	-0.880862
C	1.289368	-0.510745	-0.467043
O	-0.069807	-1.576456	1.495796
O	1.105624	0.728596	-0.611482
O	-0.269568	1.309039	1.645676
O	-2.361203	-2.653083	0.331580

O	-2.485726	-0.205270	-0.886114
O	1.894039	3.033863	0.850906
O	-2.386840	2.436457	0.143837
O	-0.062058	3.198572	-1.311504
H	2.451740	-1.732778	-1.911324
H	3.425062	-0.990124	-0.599224
H	2.531816	-2.540601	1.060865
H	1.716264	-3.480208	-0.242692
H	-0.943295	-1.977222	1.284736
H	-2.552675	0.719196	-0.580185
H	-1.536132	-0.410135	-0.984160
H	-1.836710	2.141881	0.891162
H	-1.744846	2.869308	-0.448488
H	0.220427	2.280425	-1.461055
H	0.558478	3.501451	-0.628030
H	-0.367259	0.413996	2.007116
H	0.123288	1.127992	0.756365
H	2.039268	2.233429	0.318339
H	1.271173	2.724074	1.526824
H	-2.727382	-1.785094	0.061315
H	-1.726692	-2.816716	-0.384421

TS3a: E(RM062X/6-31+G(d,p)) = -839.463877139

C	2.61233	-0.26168	1.60627
C	0.95138	0.69300	-0.66989
O	-0.02724	0.26108	-1.26469
C	3.29870	0.32027	0.36259

C	2.27060	-0.10982	-0.62110
O	2.33545	-1.11360	-1.31436
O	-0.16356	-2.56386	-1.34690
O	-1.61878	-1.91931	1.05205
O	1.00857	1.88408	-0.11776
O	-1.33800	2.99164	0.03701
O	-3.89378	-0.85899	0.55081
O	-0.02144	-0.15910	2.07100
O	-2.71920	1.05806	-0.92979
H	2.86083	-1.32446	1.70372
H	2.90722	0.27921	2.51312
H	3.34785	1.41026	0.42271
H	4.27863	-0.08673	0.08229
H	-3.24403	0.42659	-0.37998
H	-1.90281	0.58486	-1.16784
H	-3.01474	-1.31607	0.78664
H	-4.41915	-1.51445	0.08412
H	-1.50444	3.75926	-0.51754
H	-1.99004	2.27828	-0.25839
H	-0.30491	0.76178	2.06634
H	1.07302	-0.18310	1.80140
H	0.10045	2.32703	-0.11224
H	-0.09118	-1.61442	-1.55442
H	0.75949	-2.78897	-1.17631
H	-1.14864	-2.25286	0.26741
H	-1.00188	-1.23676	1.47274
